# TetR- and LysR-type transcriptional regulators mediate multilayered control of T3SS1 by *Vibrio parahaemolyticus* quorum sensing

**DOI:** 10.1128/mbio.02944-25

**Published:** 2025-11-12

**Authors:** Ce Zhang, Yu Wang, Chengan Wang, Jiaying Lu, Han Yin, Yan Shi, Zhe Zhao

**Affiliations:** 1 Jiangsu Province Engineering Research Center for Marine Bio-resources Sustainable Utilization, College of Oceanography, Hohai University 12462, Nanjing, Jiangsu, China; National University of Singapore, Singapore, Singapore

**Keywords:** *Vibrio parahaemolyticus*, quorum sensing, transcription factors, type III secretion system 1

## Abstract

The gram-negative pathogen *Vibrio parahaemolyticus* employs a sophisticated regulatory network to control virulence factors, including type III secretion system 1 (T3SS1), a key mediator of cytotoxicity. While the quorum sensing (QS) cascade LuxQ–ArcB–LuxO and master regulators AphA and OpaR are known to modulate T3SS1, the full scope of transcriptional regulation remains incompletely defined. Here, we combined the transcriptomic profiling of Δ*luxQ*, Δ*arcB*, and Δ*luxO* mutants with functional genetics to identify two novel transcription factors (TFs) that fine-tune T3SS1 activity. We demonstrate that TftR, a TetR family regulator, represses T3SS1 by binding the *opaR* promoter and enhancing OpaR production, thereby linking QS signaling to virulence suppression. Conversely, VltR, a LysR-type TF, directly activates *exsBA* transcription, independent of AphA/OpaR, to potentiate T3SS1-mediated cytotoxicity. Strikingly, VltR autoregulates its own expression, whereas TftR is reciprocally controlled by AphA (repressor) and OpaR (activator), revealing cross-talk between QS and accessory regulators. These findings delineate a multilayered regulatory hierarchy governing T3SS1, where TftR and VltR serve as opposing rheostats to calibrate virulence in response to cellular density and environmental cues. This work expands our understanding of *V. parahaemolyticus* pathogenicity and highlights potential targets for antivirulence therapies.

**IMPORTANCE:**

*Vibrio parahaemolyticus* is a major global cause of seafood-associated gastroenteritis, relying on its tightly controlled T3SS1 for virulence. While the quorum sensing regulators AphA and OpaR are known to modulate T3SS1, the full regulatory network remains incompletely understood. This study identifies two novel transcription factors, TftR (TetR family) and VltR (LysR family), that fine-tune T3SS1 activity through distinct mechanisms. These findings reveal a multilayered regulatory hierarchy that enables *V. parahaemolyticus* to precisely calibrate virulence in response to cell density and environmental cues. Understanding these regulatory interactions provides new insights into bacterial pathogenesis and may guide the development of targeted antivirulence strategies against this clinically important pathogen.

## INTRODUCTION

*Vibrio parahaemolyticus*, a gram-negative halophilic bacterium within the Vibrionaceae family, is ubiquitously distributed in estuarine, marine, and coastal ecosystems (1). As the predominant causative agent of seafood-associated gastroenteritis globally (2), this pathogen employs an extensive virulence repertoire including proteases, hemolysins, and type III secretion systems (T3SSs) (3). T3SS is a specialized secretion apparatus that functions like a molecular syringe, enabling bacteria to inject effector proteins or exotoxins directly into eukaryotic host cells (4). *V. parahaemolyticus* possesses two distinct T3SSs, each encoded on separate chromosomes (5). While type III secretion system 1 (T3SS1) modulates environmental fitness through biofilm formation, motility, and cytotoxicity, T3SS2 primarily mediates immune evasion by suppressing host inflammatory responses (1).

The transcriptional regulation of T3SS1 is governed by the *exsACDE* operon, wherein ExsA serves as the master transcriptional activator (6, 7). This regulatory cascade is initiated when ExsE secretion relieves ExsD-mediated inhibition of ExsC, ultimately enabling ExsA activation. The *exsB* gene is also located within this regulatory cluster and functions as a pilotin that promotes the assembly of the T3SS secretin in the outer membrane (8). Notably, *exsB* and *exsA* are cotranscribed, and the quorum sensing (QS) master regulators AphA and OpaR primarily regulate *exsA* through the *exsB* promoter (9, 10). Therefore, the promoter region upstream of *exsB* is the primary regulatory point for the entire ExsACDE cascade. Multiple additional regulators of *exsA* have been identified in *Vibrio* species. H-NS represses *exsA* transcription, while HlyU counteracts this repression (11, 12). The GntR family regulator SwrZ acts as a repressor of *exsA*, and the TetR-type regulator SwrT modulates *exsA* through both SwrZ-dependent and SwrZ-independent mechanisms to activate T3SS1 (13, 14). Furthermore, the two-component system VbrK/VbrR regulates T3SS1 expression via the Exs regulatory cascade (15).

The Exs regulatory cascade is intricately connected to QS networks through AphA and OpaR (16). QS represents a sophisticated cell density-dependent communication system in which autoinducer accumulation triggers population-wide gene expression changes (17). The QS system of *V. parahaemolyticus* shares a conserved core pathway with *Vibrio harveyi*, centered on the phosphorylation-dependent regulation of LuxO (18). The QS circuit integrates signals from three parallel two-component systems—LuxN, LuxPQ, and CqsS—which converge at the phosphorelay protein LuxU to modulate LuxO activity. Under low cell density (LCD), the sensor kinases LuxN, LuxPQ, and CqsS autophosphorylate and transfer phosphate groups to LuxO via LuxU. Phosphorylated LuxO activates transcription of five small regulatory RNAs (*qrr1*–*qrr5*), which post-transcriptionally regulate target mRNAs (19, 20). The Qrr sRNAs destabilize *luxR* mRNA while stabilizing *aphA* mRNA, leading to AphA accumulation and OpaR repression. At high cell density (HCD), autoinducer accumulation inhibits kinase activity, resulting in LuxO dephosphorylation. Consequently, *qrr* transcription is abolished, enabling constitutive OpaR expression, which in turn represses *aphA* (21). Functioning as master regulators that control hundreds of genes (22), AphA directly activates T3SS1 expression, whereas OpaR suppresses it (23, 24).

Recent work revealed an oxygen-sensitive regulatory branch in which ArcB replaces LuxU under aerobic conditions, phosphorylating LuxO to activate T3SS1-mediated cytotoxicity (25). Intriguingly, under hypoxia, ArcB preferentially phosphorylates its cognate partner ArcA, which appears to be dispensable for T3SS1 regulation. Despite these advances, the complete regulatory network governing T3SS1 remains incompletely characterized. Our study employs RNA sequencing to identify novel LuxQ–ArcB–LuxO pathway effectors, revealing two previously uncharacterized transcription factors: TftR (TetR family) and VltR (LysR family). The TetR family of regulators (TFRs) is one of the largest and most well-studied families of transcriptional regulators in bacteria, named after the prototype protein, TetR, which confers tetracycline resistance (26–29). TFRs are typically homodimeric repressors that control their own expression and that of an adjacent gene, often encoding an antibiotic efflux pump or an enzyme involved in detoxification (30, 31). However, the functional scope of the TetR family extends far beyond antibiotic resistance. These regulators are involved in a stunning array of critical physiological functions, such as biosynthesis of antibiotics, catabolism of aromatic compounds, quorum sensing, stress responses, and virulence (32, 33). The LysR-type transcriptional regulators (LTTRs) constitute the largest family of prokaryotic regulators, with members found in nearly all bacterial species (34–36). Unlike many TetR proteins, LTTRs are typically transcriptional activators, though they often autorepress their own expression (37). Their activation mechanism often involves a coinducer molecule that binds to the regulator, altering its interaction with RNA polymerase to stimulate transcription of the target genes (38, 39). LTTRs are master regulators of genes involved in basic metabolism but also influence key adaptive and virulence phenotypes such as virulence, biofilm formation, motility, quorum sensing, antibiotic resistance, and stress tolerance (37, 40).

The TetR and LysR families represent two pillars of bacterial transcriptional regulation. They exemplify the sophisticated mechanisms bacteria have evolved to thrive in dynamic environments. Here, we demonstrate that two new regulatory factors from the TetR and LysR families regulate the T3SS1 of *V. parahaemolyticus* through different mechanisms. TftR modulates T3SS1 through OpaR activation, whereas VltR directly enhances *exs* operon transcription. This multilayered regulatory architecture enables *V. parahaemolyticus* to precisely calibrate T3SS1 activity, optimizing virulence expression while minimizing fitness costs.

## RESULTS

### Identification of QS-dependent transcriptional regulators controlling T3SS1 in *V. parahaemolyticus*

**Fig 1:**
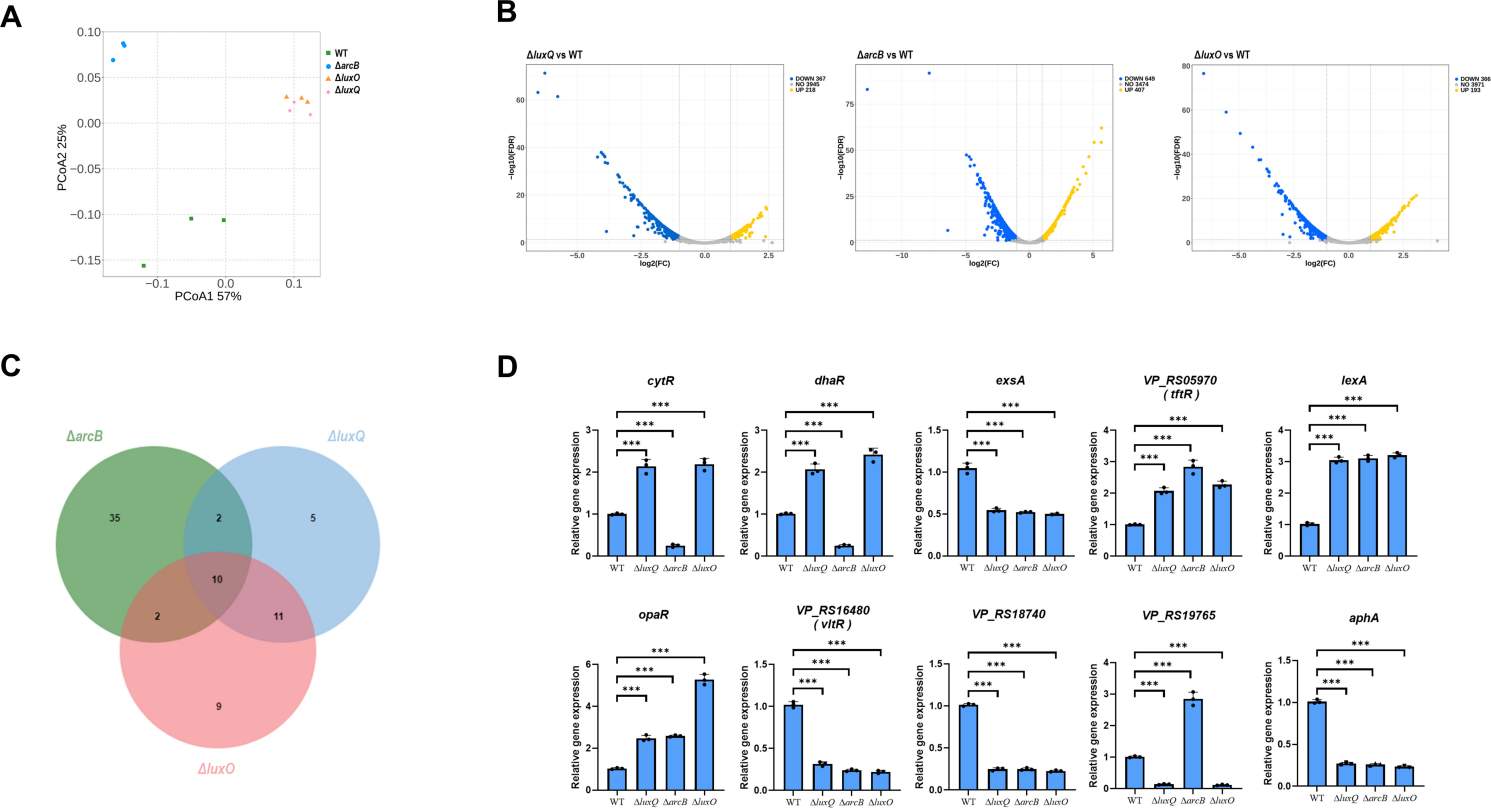
Identification of QS-dependent transcriptional regulators controlling T3SS1 in *V. parahaemolyticus*. (**A**) Principal coordinate analysis (PCoA) plot based on Bray–Curtis dissimilarity. Sample points are colored/shaped by WT, Δ*arcB*, Δ*luxO*, and Δ*luxQ*. Axes show principal coordinates (PCoA1 and PCoA2). (**B**) Volcano plot of differential expression between the WT and mutant strains. (**C**) Venn diagram showing the number of exclusive and shared differentially expressed transcription factors in different strains. (**D**) Transcript levels of the indicated genes in the WT, Δ*luxQ*, Δ*arcB*, and Δ*luxO* strains were measured via quantitative real-time PCR. The data were normalized to *rpoA* expression. Data are presented as means ± SD from three independent experiments. Statistical significance was determined using one-way analysis of variance. ***P < 0.0005.

To elucidate the transcriptional regulatory network governing T3SS1 in *V. parahaemolyticus*, we conducted comprehensive RNA-seq analysis of wild-type (WT) and isogenic mutant strains (Δ*luxQ*, Δ*arcB*, and Δ*luxO*) under T3SS1-inducing conditions. Principal coordinate analysis revealed striking similarities between Δ*luxQ* and Δ*luxO* mutants, reflecting their established hierarchical relationship in the QS cascade where LuxQ acts upstream of LuxO. In contrast, the Δ*arcB* mutant exhibited a distinct transcriptional profile, consistent with its broader role in integrating diverse environmental signals beyond QS (Fig. 1A). Global transcriptomic changes (fold change >2, false discovery rate [FDR] <0.05) were most pronounced in Δ*arcB* (649 upregulated and 407 downregulated genes), followed by Δ*luxQ* (218 upregulated and 367 downregulated) and Δ*luxO* (193 upregulated and 366 downregulated) (Fig. 1B), demonstrating the extensive regulatory influence of this pathway. Gene Ontology (GO) analysis of differentially expressed genes (DEGs) highlighted significant enrichment in functional categories, including small molecule catabolic processes, cell motility, and transmembrane transport (Fig. S1), suggesting that disruption of QS and ArcB triggers not only virulence-related changes but also broad metabolic and physiological adaptations.

Through integrative analysis of the RNA-seq data sets, we identified a core set of transcription factors that are coordinately regulated by the LuxQ–ArcB–LuxO signaling pathway (Fig. 1C). Specifically, 28 transcription factors showed *luxQ*-dependent regulation; 49 were responsive to *arcB*; and 32 exhibited *luxO*-mediated expression changes, with 10 consistently coregulated factors that may represent key nodes in the T3SS1 regulatory network. Validation by quantitative real-time PCR (qRT-PCR) revealed two distinct regulatory patterns (Fig. 1D): *VP_RS05970* (a TetR family regulator), *lexA* (SOS response regulator), and *opaR* (quorum sensing master regulator) were significantly upregulated in all the mutant strains, whereas *exsA* (T3SS1 transcriptional activator), *VP_RS16480* (LysR family regulator), *VP_RS18740* (LysR family regulator), and *aphA* (quorum sensing regulator) were markedly downregulated. These findings not only confirm the expected reciprocal regulation of AphA and OpaR within the quorum sensing circuitry but also reveal novel candidate regulators for integration with the established T3SS1 regulatory network.

### TftR and VltR are involved in the regulation of T3SS1

**Fig 2:**
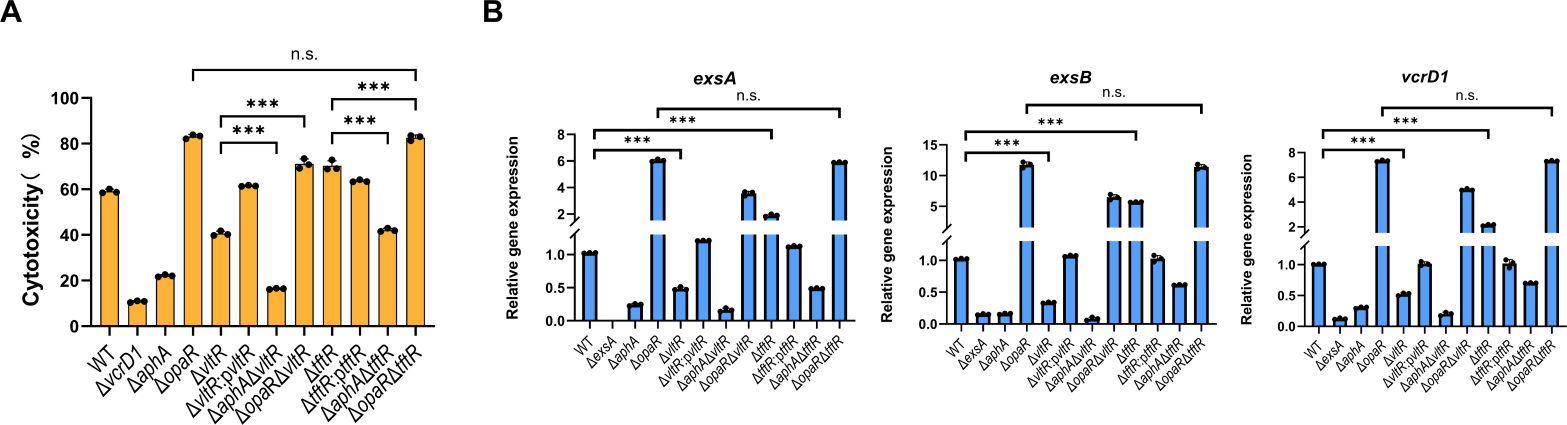
TftR and VltR are involved in the regulation of T3SS1. (**A**) Lactate dehydrogenase assay results (% cytotoxicity) for HeLa cells infected with different strains, including WT strain, indicated mutant strains, and complementation strains. The pBBR1MCS-1 plasmid was used for all complemented strains. Data are presented as means ± SD from three independent experiments. Statistical significance was determined using one-way analysis of variance (ANOVA). ***P < 0.0005. n.s., not significant. (**B**) Transcriptional levels of the T3SS genes *exsA*, *exsB*, and *vcrD1* (structural protein-encoding gene) in the indicated strains were determined via qRT-PCR. The data were normalized to *rpoA* expression. Data are presented as means ± SD from three independent experiments. Statistical significance was determined using one-way ANOVA. ***P < 0.0005. n.s., not significant.

The functional characterization of VP_RS05970 (now designated as TftR for TetR-family T3SS1 regulator) and VP_RS16480 (now designated as VltR for *Vibrio* LysR-type T3SS1 regulator) in T3SS1 regulation revealed their opposing roles in virulence control. Cytotoxicity assays showed that, compared to the WT strain, the Δ*aphA* and Δ*vltR* mutants exhibited significantly reduced cytotoxicity, whereas the Δ*opaR* and Δ*tftR* mutants displayed enhanced cytotoxicity (Fig. 2A). Notably, the effects of *vltR*, *aphA*, and *opaR* on cytotoxicity were additive, but the regulatory effects of AphA and OpaR are stronger than those of VltR. The phenotypic similarity between the *opaR* single mutant and *tftR opaR* double mutant suggests that OpaR is epistatic to Tftr (Fig. 2A).

Corresponding transcriptional analyses further demonstrated that VltR acts as a positive regulator of T3SS1 components, with the Δ*vltR* mutant showing downregulation of *exsA*, *exsB*, and *vcrD1* expression (Fig. 2B). Conversely, TftR acts as a repressor, as evidenced by the upregulation of these genes in Δ*tftR*. Furthermore, the level of activation provided by VltR is independent, but the regulatory effect is less than that of AphA/OpaR. However, TftR-mediated regulation of T3SS1-related genes is OpaR dependent (Fig. 2B). The magnitude of the transcriptional changes correlated well with the observed cytotoxicity phenotypes, suggesting that these regulators primarily affect T3SS1 activity through transcriptional control rather than posttranslational mechanisms.

### VltR directly activates T3SS1 through the *exs* operon

**Fig 3:**
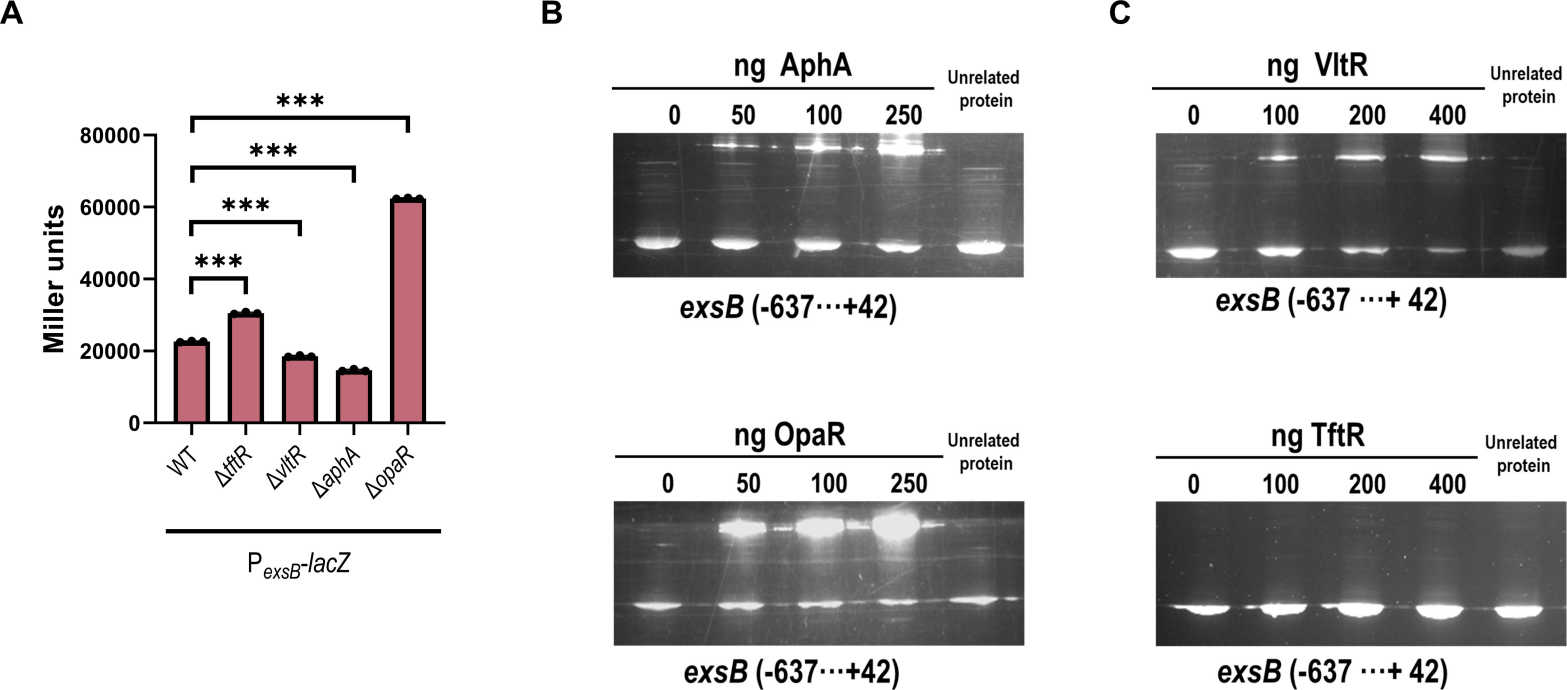
VltR directly activates T3SS1 through the *exs* operon. (**A**) LacZ fusion. The promoter-proximal DNA region of exsB was cloned and inserted into the pHRP309 vector and then transferred into the WT and each mutant strain to determine the promoter activity, that is, the β-galactosidase activity (Miller units) in the cellular extracts. Data are presented as means ± SD from three independent experiments. Statistical significance was determined using one-way ANOVA. ***P < 0.0005. (**B and C**) Electrophoretic mobility shift assay. The promoter-proximal DNA fragments of *exsB* were incubated with increasing amounts of purified proteins and then subjected to 8% (wt/vol) polyacrylamide gel electrophoresis. As a control, 400 ng of the unrelated protein LuxP was used to replace the target protein in the reaction. Data shown represent one of three independent experiments with similar results.

We want to test whether TftR and VltrR regulate T3SS1 through the Exs regulatory cascade. As the promoter upstream of *exsB* is responsible for driving the expression of the *exsBA* operon (and consequently the master regulator ExsA), we chose *exsB* as the target gene for promoter activity assays and DNA-binding studies. P*_exsB_*-lacZ reporter assays revealed that *exsB* promoter activity in Δ*vltR* was lower than that in WT, whereas Δ*tftR* presented greater activity than WT, which is consistent with their respective roles as activators and repressors of T3SS1 (Fig. 3A).

Electrophoretic mobility shift assays (EMSAs) revealed distinct DNA-binding properties of these regulators (Fig. 3B and C). VltR, along with the known regulators AphA and OpaR, bound specifically to the *exsB* promoter region in a concentration-dependent manner. In contrast, TftR showed no detectable binding to this promoter. Furthermore, the inclusion of an unrelated protein in the reactions verified that the observed gel shifts were dependent on the specific regulators and not due to non-specific protein interactions. These findings indicate that VltR directly activates T3SS1 expression by binding to the *exs* operon promoter, whereas TftR likely exerts its regulatory effects through an indirect mechanism.

### TftR inhibits the expression of the T3SS1 gene by activating OpaR

**Fig 4:**
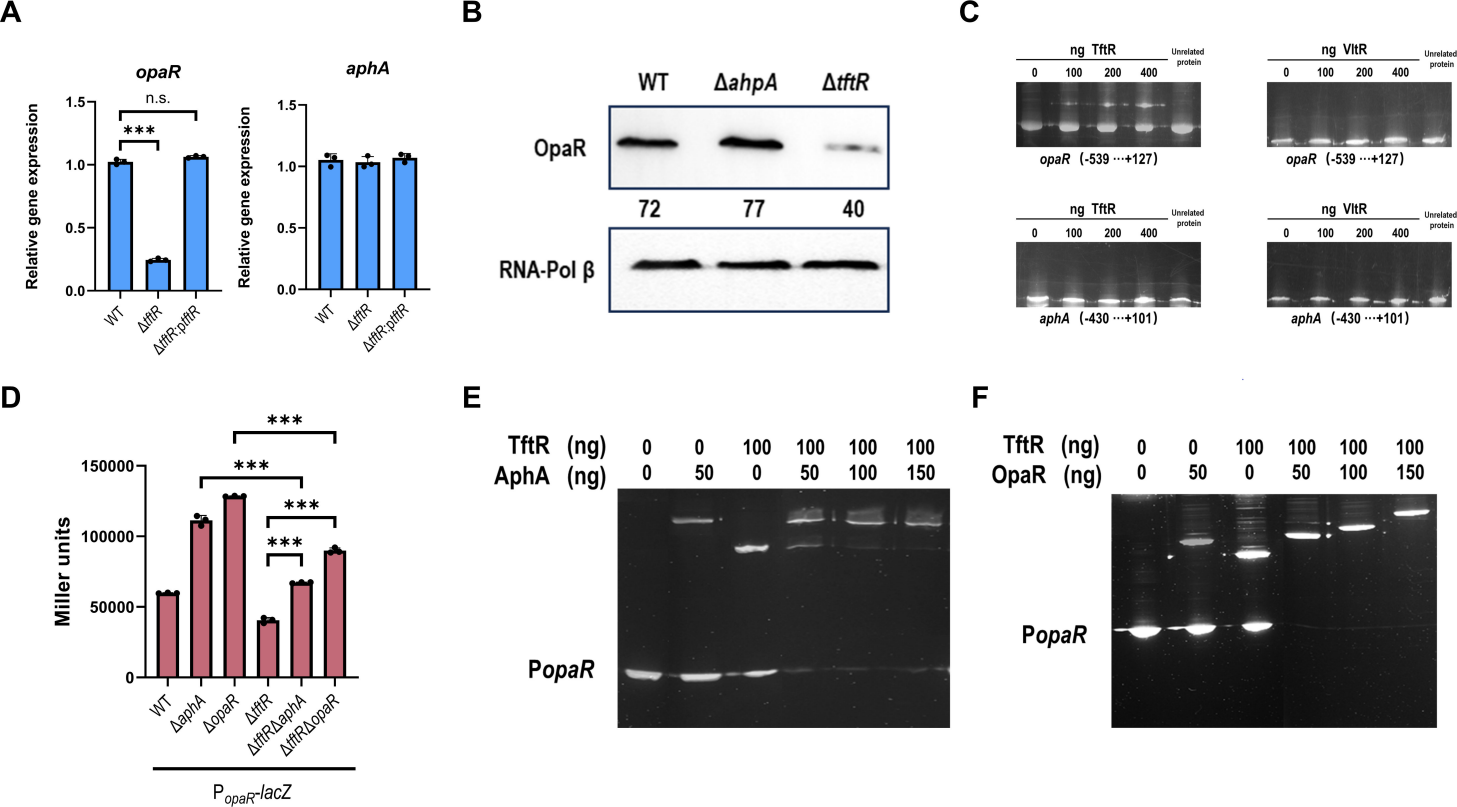
TftR inhibits the expression of the T3SS1 gene by activating *opaR*. (**A**) Transcriptional levels of *opaR* and *aphA* in the indicated strains determined via qRT-PCR. The data were normalized to rpoA expression. Data are presented as means ± SD from three independent experiments. Statistical significance was determined using one-way ANOVA. ***P < 0.0005. n.s., not significant. (**B**) Western blot analysis of OpaR expression in WT, Δ*aphA*, and Δ*tftR* cells. RNA-Pol β was used as a loading control for data normalization, and quantification was performed using Image J software. Data shown represent one of three independent experiments with similar results. (**C**) EMSA. The promoter-proximal DNA fragments of the *opaR* or *aphA* gene were incubated with increasing amounts of purified proteins and then subjected to 8% (wt/vol) polyacrylamide gel electrophoresis. As a control, 400 ng of the unrelated protein LuxP was used to replace the target protein in the reaction. Data shown represent one of three independent experiments with similar results. (**D**) LacZ fusion. The promoter-proximal DNA region of *opaR* was cloned and inserted into the pHRP309 vector and then transferred into the WT and mutant strains to determine the promoter activity, that is, the β-galactosidase activity (Miller units) in the cellular extracts. Data are presented as means ± SD from three independent experiments. Statistical significance was determined using one-way ANOVA. ***P < 0.0005. (**E and F**) EMSA. The promoter-proximal DNA fragments of *opaR* were incubated with increasing amounts of purified proteins and then subjected to 8% (wt/vol) polyacrylamide gel electrophoresis. Data shown represent one of three independent experiments with similar results.

Next, we further investigated how TftR regulates T3SS1. Given that *V. parahaemolyticus* T3SS1 is controlled by the QS master regulators AphA and OpaR, we examined whether TftR regulates T3SS1 by affecting the QS master regulator. qRT-PCR experiments revealed a significant reduction in *opaR* transcript levels in the Δ*tftR* mutant compared with both the WT and complemented strains (Δ*tftR*:p*tftR*), whereas *aphA* expression remained unaffected (Fig. 4A). This transcriptional regulation was further confirmed at the protein level through Western blot analysis, which demonstrated substantially decreased OpaR production in the Δ*tftR* strain relative to that in the WT strain (Fig. 4B). These findings establish that TftR specifically activates *opaR* expression without influencing *aphA* transcription, thereby positioning it as a novel component in the QS regulatory cascade controlling T3SS1.

To molecularly characterize the interaction between TftR and the *opaR* promoter, we performed EMSA with purified TftR protein. The results demonstrated concentration-dependent binding of TftR to the P*opaR* DNA fragment, with increasing protein concentrations (0–400 nM), resulting in progressive retardation of DNA probe migration (Fig. 4C). Importantly, control experiments revealed no detectable binding of TftR to the *aphA* promoter (P*_aphA_*), confirming its binding specificity. Furthermore, parallel EMSA with VltR revealed no interaction with either P*_opaR_* or P*_aphA_*. These data confirm that TftR specifically recognizes and binds to the *opaR* promoter region to regulate its expression.

In *V. parahaemolyticus*, the *opaR* promoter is known to be regulated through complex interactions involving AphA binding and OpaR autoregulation. Our genetic analysis using a P*opaR*-lacZ reporter system revealed an intricate regulatory relationship between TftR and these established regulators. The double mutant Δ*tftR*Δ*aphA* presented intermediate P*opaR* activity levels, which were significantly greater than those of Δ*tftR* alone but lower than those of Δ*aphA* (Fig. 4D). Similarly, P*opaR* activity in Δ*tftR*Δ*opaR* was greater than that in Δ*tftR* but lower than that in Δ*opaR*, suggesting that TftR functions in parallel with both AphA and OpaR in regulating *opaR* expression. EMSAs revealed that although TftR or AphA forms discrete protein–DNA complexes with P*opaR* alone, an increase in the AphA concentration (0–150 nM) gradually replaces the prebound TftR from the promoter (Fig. 4E). In contrast, when OpaR was titrated against constant TftR concentrations, we observed a progressive decrease in free promoter fragments and the formation of higher-molecular-weight complexes (Fig. 4F). These findings establish that TftR integrates into the existing QS regulatory network by binding to the *opaR* promoter.

### VltR and TftR have different regulatory mechanisms

**Fig 5:**
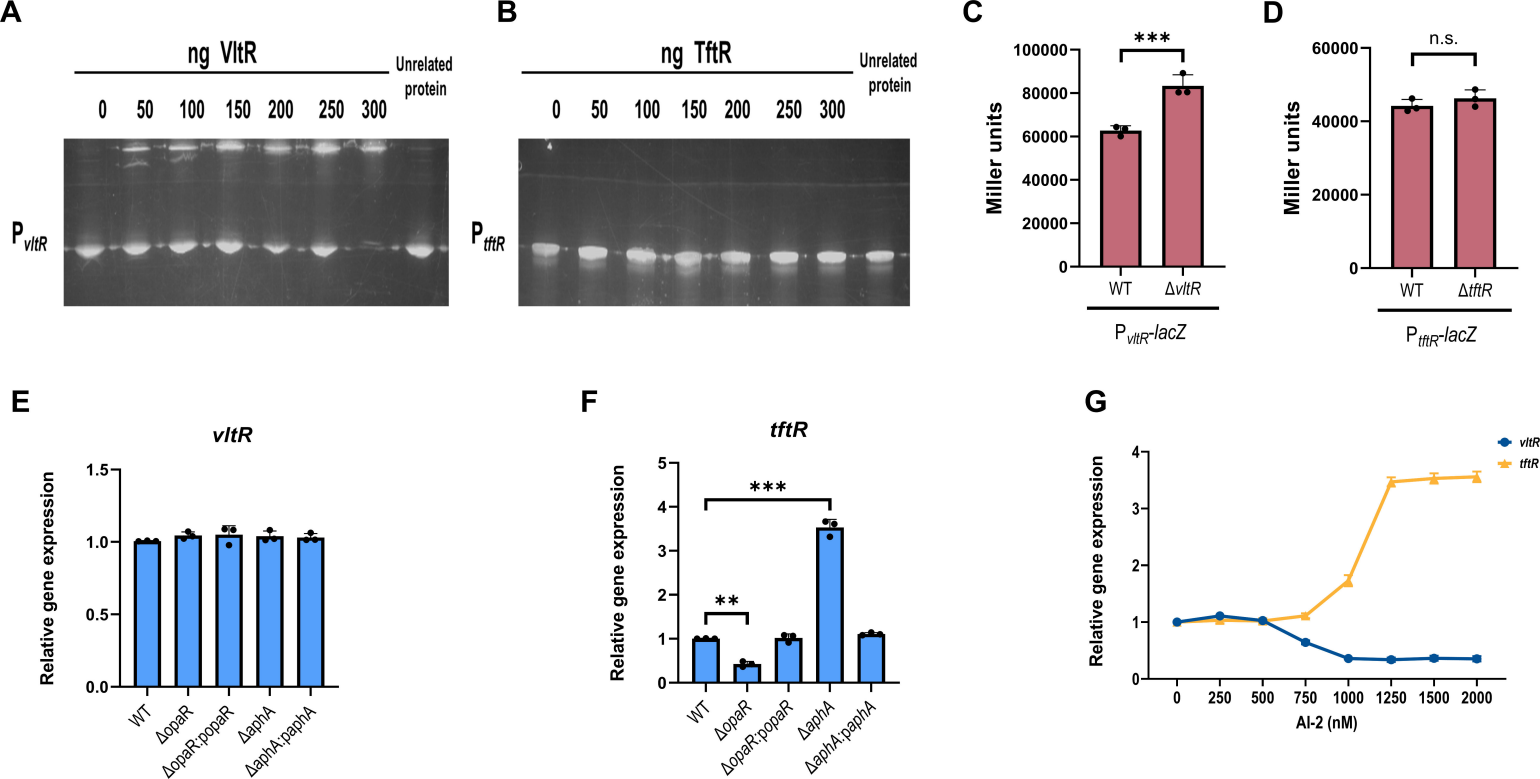
VltR and TftR have different regulatory mechanisms. (**A and B**) EMSA. The promoter-proximal DNA fragments of *vltR* or *tftR* were incubated with increasing amounts of purified proteins and then subjected to 8% (wt/vol) polyacrylamide gel electrophoresis. As a control, 400 ng of the unrelated protein LuxP was used to replace the target protein in the reaction. Data shown represent one of three independent experiments with similar results. (**C and D**) LacZ fusion. The promoter-proximal DNA region of the target gene was cloned and inserted into the pHRP309 vector and then transferred into the WT and each mutant strain to determine the promoter activity, that is, the β-galactosidase activity (Miller units) in the cellular extracts. Data are presented as means ± SD from three independent experiments. Statistical significance was determined using unpaired two-tailed *t*-test. ***P < 0.0005. n.s., not significant. (**E and F**) Transcriptional levels of the target genes in the indicated strains were determined via qRT-PCR. The data were normalized to *rpoA* expression. The pBBR1MCS-1 plasmid was used for all complemented strains. Data are presented as means ± SD from three independent experiments. Statistical significance was determined using one-way ANOVA. **P < 0.005, ***P < 0.0005. (**G**) Transcriptional levels of *vltR* and *tftR* in the *V. parahaemolyticus* cells grown under various concentrations of AI-2. The data were normalized to rpoA expression. Data are presented as means ± SD from three independent experiments.

To investigate the autoregulatory capacity of VltR and TftR, we performed EMSAs using purified proteins and their respective promoter regions. The EMSA results demonstrated that VltR bound to its own promoter (P*_vltR_*) in a concentration-dependent manner, with increasing protein concentrations (0–300 nM) resulting in progressive DNA retardation (Fig. 5A). In contrast, TftR showed no detectable binding to its cognate promoter (P*_tftR_*), even at the highest concentration tested (Fig. 5B). To determine the functional consequences of these DNA-binding activities, we constructed lacZ transcriptional fusions and measured promoter activity in relevant genetic backgrounds. Compared with the WT, the P*_vltR_*-lacZ reporter exhibited significantly greater activity in the Δ*vltR* mutant (Fig. 5C), demonstrating that VltR functions as an autorepressor. Conversely, the activity of the P*_tftR_*-lacZ reporter did not significantly differ between the WT and Δ*tftR* strains (Fig. 5D), confirming the absence of autoregulation of TftR.

To further characterize the regulatory mechanisms controlling VltR and TftR expression, we performed qRT-PCR analysis of the WT, Δ*opaR*, Δ*aphA*, and complemented strains. The results revealed that the VltR transcript levels remained unchanged across all the genetic backgrounds (Fig. 5E). In contrast, TftR expression was clearly dependent on QS regulators. The transcript level of *tftR* decreased in the Δ*opaR* strain compared with those in the WT strain and was restored in the complemented strain, whereas it increased in the Δ*aphA* strain and returned to WT levels in the complemented strain (Fig. 5F). To investigate the production of VltR and TftR during quorum sensing mediated by the LuxQ–ArcB–LuxO pathway, we measured the relative production of *vltR* and *tftR* transcripts at different AI-2 concentrations. AI-2 is sensed by the membrane-bound receptor LuxQ, which then transmits the signal through the ArcB–LuxO pathway to regulate downstream gene expression. We found that as the concentration of AI-2 increased; *vltR* levels decreased; and *tftR* levels increased (Fig. 5G). Therefore, the expression of VltR and TftR is cell density dependent, which is consistent with the density-dependent regulation of T3SS1. These findings demonstrate that TftR is positively regulated by OpaR and negatively regulated by AphA. The expression of VltR is not affected by these QS regulatory factors but is directly regulated by the quorum sensing signals sensed and transmitted by LuxQ. The different regulation of these two transcription factors reveals their distinct roles in the regulatory network. TftR works synergistically with QS regulatory factors, while Vltr operates independently.

## DISCUSSION

T3SSs serve as a crucial virulence determinant in pathogenic *Vibrio* species, mediating the direct translocation of effector proteins into host cells to manipulate cellular processes (1). In *V. parahaemolyticus*, T3SS1 expression is controlled through the LuxQ‒ArcB‒LuxO pathway, with the master regulators AphA and OpaR orchestrating opposing transcriptional responses at LCD and HCD via direct regulation of the *exs* operon. The complexity of QS-mediated virulence regulation is evidenced by comparative genomic studies demonstrating that while OpaR controls approximately 5.2% of the *V. parahaemolyticus* genome (23) and that HapR regulates 4.1% of *V. cholerae* (19), substantial portions of the QS regulon remain unaccounted for, suggesting the involvement of additional regulatory components. Our systematic approach combining RNA-seq analysis of Δ*luxQ*, Δ*arcB*, and Δ*luxO* mutants with functional validation identified seven transcription factors coregulated by the LuxQ–ArcB–LuxO pathway. Among these, TftR (TetR family) and VltR (LysR family) have emerged as novel T3SS1 modulators. These findings expand our understanding of the sophisticated regulatory network governing *V. parahaemolyticus* virulence, revealing how accessory transcription factors may fine-tune T3SS1 activity alongside the core QS circuitry.

Our study identified TftR as a TFR that modulates T3SS1 expression in *V. parahaemolyticus* through an OpaR-dependent mechanism. TFRs are characterized by an N-terminal helix-turn-helix (HTH) DNA-binding domain and a C-terminal dimerization/ligand-binding domain, enabling diverse regulatory functions (41). While classical TetR proteins function primarily as transcriptional repressors, our findings reveal that TftR participates in a more complex regulatory cascade. EMSAs revealed that TftR directly binds the *opaR* promoter, suggesting its role in *opaR* transcriptional regulation. Intriguingly, TftR functions in concert with the master QS regulators AphA and OpaR, forming a multilayered regulatory network. Both AphA and OpaR have been identified as repressors of *opaR* expression (42, 43). An AphA box-like sequence overlaps with the −10 core promoter region, while an OpaR box-like sequence is located downstream of the transcription start site. Direct binding of AphA and OpaR to the *opaR* promoter impedes RNA polymerase access, thereby inhibiting *opaR* transcription. Using an EMSA probe containing both AphA and OpaR binding sites, we observed that increasing concentrations of AphA compete with TftR for probe binding. Furthermore, in the presence of both OpaR and TftR, a DNA supershift occurred. These results indicate that all three transcription factors can bind simultaneously to the P*_opaR_* region, with OpaR and TftR likely exhibiting cooperative binding. TftR expression is reciprocally regulated—positively by OpaR and negatively by AphA—creating a coherent feed-forward loop that reinforces OpaR-mediated T3SS1 repression at HCD (21). This regulatory interplay allows *V. parahaemolyticus* to fine-tune T3SS1 activity during infection. At LCD, AphA dominates, suppressing TftR while activating T3SS1 to promote early-stage cytotoxicity (42). Under HCD, OpaR accumulates, increasing TftR expression and further amplifying OpaR-mediated T3SS1 repression, facilitating intestinal colonization. Thus, TftR serves as a critical node in the QS–T3SS1 regulatory network, enabling precise spatiotemporal control of virulence gene expression. This sophisticated regulatory architecture may enhance bacterial adaptability during infection, balancing cytotoxicity and immune evasion strategies.

The T3SS is strictly controlled by the protein encoded by the regulatory operon Exs, especially the central positive regulatory factor ExsA (44). In this study, we confirmed the specific binding of VltR to the P*_exsB_* promoter region *in vitro* and activated the promoter activity of P*_exsB_ in vivo*. Therefore, VltR directly participates in the transcription of ExsBA, indicating that the T3SS1 of *V. parahaemolyticus* is positively controlled by VltR. However, we cannot rule out the possibility that there may be additional VltR binding sites upstream or internally of the *exs* operon; i.e., VltR may interact with multiple sites to control the transcription of *exsACDE*. VltR is an LTTR. LTTRs are involved in a wide range of cellular processes, including the regulation of operons responsible for various metabolic pathways, responses to environmental changes, and virulence factors in bacteria (45–47). Members of this family typically act as activators or repressors and are characterized by HTH motifs that bind to DNA (37, 48). Most LTTRs act as transcriptional activators of target genes and repressors of their own expression (49). To investigate the self-inhibitory effect of VltR, EMSA was first performed, and the results revealed that VltR directly binds to its own promoter. In addition, with the deletion of the *vltR* gene, the activity of P*_vltR_* -lacZ in cells significantly increased, indicating the negative regulation of *vltR* expression by VltR, which is a typical automatic regulatory feature of LTTR. These comprehensive data indicate that the main effect of VltR on T3SS1 gene expression is independent of AphA and OpaR but directly affects the P*_exsB_* promoter.

The observed changes in the transcriptional levels of *vltR* and *tftR* themselves and their effects on the transcriptional changes of key regulators such as *exsA* and *opaR*, although statistically significant, were generally modest (<2-fold). This is powerfully demonstrated by our phenotypic data: the deletion of *vltR* or *tftR* resulted in substantial and reproducible changes in cytotoxicity (Fig. 2), despite the modest change in their own transcript levels. This amplification effect is a classic feature of regulatory networks. We posit that the modest fold changes suggest VltR and TftR act as fine-tuning modulators within the established T3SS1 regulatory circuit, rather than as dominant on/off switches. Their role appears to be the precise adjustment of gene expression under the T3SS1-inducing conditions used here (25).

**Fig 6:**
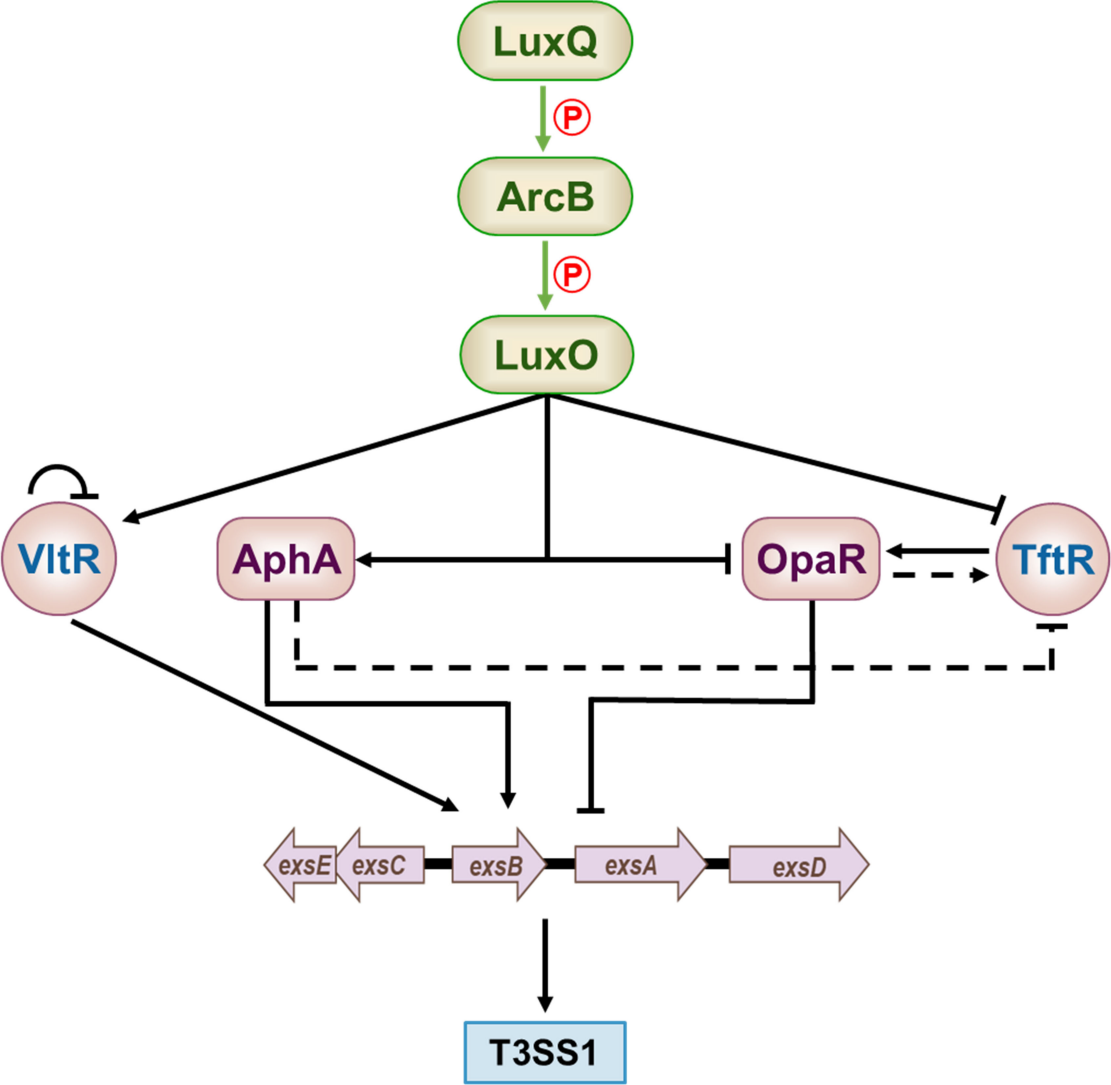
Regulatory network of T3SS1 in *V. parahaemolyticus*. Green arrows depict phosphotransfer in the LuxQ–ArcB–LuxO cascade. Solid black lines indicate direct transcriptional regulation, while dashed black lines denote indirect regulation. Arrows represent activation, and T-bars indicate repression.

Although T3SS gene expression has adaptive advantages under certain conditions, it may be disadvantageous under many other conditions because of energy and other factors. Therefore, the ability to precisely adjust the T3SS to cope with environmental changes is very important. Here, we report two novel T3SS1 regulatory factors, TftR and VltR. Although both are regulated by the upstream LuxQ–ArcB–LuxO pathway of QS, they regulate T3SS1 through different modes of action (Fig. 6). TftR negatively regulates T3SS1 by activating *opaR*. VltR can directly activate T3SS1 at the *exs* promoter level. Virulence is a complex, multilayered process controlled by numerous integrated signals. The new regulator is a new piece of this complex puzzle, which could lead to a better understanding of its virulence, survival, and adaptation mechanisms. This knowledge may also provide potential targets for the development of new antibacterial agents and control measures.

## MATERIALS AND METHODS

### Bacterial strains, plasmids, and growth conditions

The *V. parahaemolyticus* strain RIMD2210633 and its derivative mutants were routinely cultured in heart infusion (HI) broth (BD Diagnostics, USA) supplemented with 1% (wt/vol) NaCl at 37°C or on thiosulfate citrate bile salt sucrose (Huankai Microbial, China) agar plates. *Escherichia coli* strains DH5α and S17-1 were used for genetic manipulation and conjugation assays, respectively. The suicide plasmid pDM4 served as the backbone for gene knockout construction, whereas the pMMB207 and pBBR1MCS1 expression vectors were employed for complementation studies. For protein expression and purification, the pCold I vector (Takara, Japan) was utilized. The antibiotic concentrations used for selection were as follows: ampicillin (100 µg/mL) or chloramphenicol (5 µg/mL) for *V. parahaemolyticus* and ampicillin (100 µg/mL), chloramphenicol (25 µg/mL), or kanamycin (50 µg/mL) for *E. coli*. Complete strain and plasmid information is provided in Table S1.

### Construction of gene deletion mutants and complementation strains

In-frame deletion mutant strains were constructed via specific primers (Table S2) and isoallelic exchange as described previously. In brief, overlap extension (SOE) PCR was used to generate DNA fragments for in-frame deletions. These fragments were then cloned and inserted into the XbaI-digested suicide vector pDM4 via the ClonExpress II One-Step Cloning Kit (Vazyme, China) and transformed into *Escherichia coli* DH5α cells. After sequencing, the resulting plasmid was transformed into *E. coli* S17 and then mated with *V. parahaemolyticus* by conjugation. Transconjugants were selected on medium containing chloramphenicol, followed by counterselection on Luria–Bertani (LB) plates containing 10% (wt/vol) sucrose. The production of targeted mutants was confirmed via PCR. To construct complement strains, genes spanning the predicted promoter region and open reading frame (ORF) were cloned and inserted into the pBBR1MCS-1 plasmid. The derived plasmids were coupled to the relevant mutant strains. For Western blot analysis, the ORF of each gene was expressed with a C-terminal Flag tag under the control of the *tac* promoter in the pMMB207 vector.

### Cell culture and cytotoxicity assay

HeLa cells were maintained in Dulbecco’s modified Eagle’s medium (DMEM; Gibco, USA) supplemented with 10% fetal bovine serum (Gibco) and 1% penicillin/streptomycin (Gibco) at 37°C in a humidified 5% CO_2_ atmosphere. For the cytotoxicity assays, the cells were seeded at a density of 1 × 10^5^ cells/well in 96-well tissue culture plates and allowed to adhere for 12 h prior to infection. Overnight cultures of *V. parahaemolyticus* strains were subcultured 1:100 in fresh HI medium and grown to mid-exponential phase (OD_600_ = 0.5) at 37°C with shaking (200 rpm). The cell monolayers were washed twice with phosphate-buffered saline, and the medium was replaced with serum-free DMEM. Bacteria were added at a multiplicity of infection of 20:1. Infected cells were incubated for 2 h at 37°C. Cytotoxicity was quantified via the Lactate Dehydrogenase Cytotoxicity Detection Kit (Roche Diagnostics, Germany) according to the manufacturer’s protocol.

### Protein expression and purification

The ORFs of the target genes were amplified via PCR via gene-specific primers (Table S2). Each ORF was subsequently cloned and inserted into the pCold I expression vector to generate C-terminal 6× His-tagged fusion constructs. The recombinant plasmids were transformed into *E. coli* BL21(DE3) competent cells for protein expression. Starter cultures were grown overnight at 37°C in LB media supplemented with 100 µg/mL ampicillin. Cultures were inoculated at a ratio of 1:100 and grown at 37°C with shaking (220 rpm) until the OD_600_ reached 0.5. The cultures were rapidly cooled to 20°C in an ice–water bath. Protein expression was induced with 0.5 mM isopropyl β-D-1-thiogalactopyranoside. The induced cultures were incubated at 20°C for 20 h with continuous shaking (100 rpm). Finally, the proteins were purified via nickel affinity chromatography and concentrated via an Amicon Ultracentrifugation Filter (Millipore, USA). The protein concentration was determined via the Bradford assay. Purity was verified by SDS‒PAGE and Coomassie blue staining.

### Western blot analysis

Whole-cell protein was obtained via BugBuster Master Mix, and protein concentrations were subsequently determined via a Bio-Rad protein assay kit. Equal amounts of proteins from different *V. parahaemolyticus* strains were separated by SDS‒PAGE, subsequently transferred to polyvinylidene difluoride membranes, and blocked in 5% skim milk overnight at 4°C. The membranes were incubated with the primary antibody for 2 h at room temperature, followed by incubation with the secondary antibody for 1 h. The blots were visualized via SuperSignal West Pico PLUS chemiluminescence substrate (Thermo Fisher Scientific, USA) and quantified with ImageJ software.

### EMSA

The gel shift assay was performed via an EMSA Kit (Invitrogen) according to the manufacturer’s instructions. Briefly, target DNA was incubated with increasing amounts of protein extract for 20 min at room temperature in a 20 µL reaction mixture containing 1× binding buffer (750 mM KCl, 0.5 mM dithiothreitol, 0.5 mM EDTA, 50 mM Tris-HCl, pH 7.4). As a control, 400 ng of the unrelated protein LuxP was used to replace the target protein in the reaction. Following the addition of EMSA gel-loading solution, the mixtures were separated via electrophoresis on a nondenaturing 8% (wt/vol) polyacrylamide gel in 0.5× TBE buffer (22 mM Tris-HCl, 22 mM boric acid, 0.5 mM EDTA, pH 8.0), and the gels were stained with 1× SYBR Green EMSA nucleic acid stain. The DNA was then visualized via digital photography with a SYPRO photographic filter.

### Transcriptional profiling and bioinformatics analysis

The cultivation of the WT, Δ*luxQ*, Δ*arcB*, and Δ*luxO* strains in HI medium was repeated until the OD_600_ = 0.6. The culture mixture was centrifuged; the pellet was resuspended in a 12 mL loose cap culture tube with 3 mL of DMEM; and the mixture was shaken at 200 rpm for 3 h at 37°C. Total RNA was extracted from the tissue via TRIzol Reagent according to the manufacturer’s instructions (Invitrogen), and genomic DNA was removed via DNase I (Takara). The RNA-seq strand-specific libraries were prepared with a TruSeq RNA sample preparation kit from Illumina (San Diego, CA) with 5 µg of total RNA. After quantification by TBS380, paired-end libraries were sequenced via Illumina NovaSeq 6000 sequencing (150 bp*2, Shanghai BIOZERON Co., Ltd.). The raw paired-end reads were trimmed and quality controlled by Trimmomatic with parameters (SLIDINGWINDOW:4:15 MINLEN:75). The clean reads were subsequently separately aligned to the reference genome in orientation mode via Rockhopper (https://cs.wellesley.edu/~btjaden/Rockhopper/) software, which was subsequently used to calculate gene expression levels. To identify DEGs between the two different samples, the expression level of each transcript was calculated via the fragments per kilobase of reads per million mapped reads method. edgeR (https://bioconductor.org/packages/release/bioc/html/edgeR.html) was used for differential expression analysis. The DEGs between two samples were selected according to the following criteria: (i) the logarithm of the fold change was greater than 2, and (ii) the FDR was less than 0.05. To understand the functions of the DEGs, GO functional enrichment and Kyoto Encyclopedia of Genes and Genomes pathway analyses were carried out via Goatools (https://github.com/tanghaibao/Goatools) and KOBAS (http://bioinfo.org/kobas), respectively. DEGs were significantly enriched in GO terms and metabolic pathways when their Bonferroni-corrected *P* value was less than 0.05.

### LacZ reporter system and β-galactosidase activity assay

The regulatory DNA region of each target gene was cloned and inserted into the lacZ reporter vector pHRP309, which carries a promoterless *lacZ* gene and a gentamicin resistance marker. The resulting recombinant plasmids were then introduced into both the WT strain and its corresponding mutant strain. The bacterial cultures were grown in HI media at 37°C for 3 h, after which the cells were harvested and lysed. β-Galactosidase activity was subsequently measured in the supernatants of the bacterial lysates.

### qRT-PCR

qRT‒PCR was performed via an Applied Biosystems 7500 Real-Time PCR System under the following conditions: initial denaturation at 95°C for 30 s, followed by 40 cycles at 95°C for 10 s and 60°C for 30 s. The mRNA expression levels of target genes were normalized to those of the reference gene *rpoA*, and relative quantification was determined via the ΔΔCt method, with WT samples set as the baseline. All reactions were conducted in triplicate. The primer sequences for the target and reference genes, which were designed via National Center for Biotechnology Information tools, are listed in Table S2.

### Statistical analysis

Data were presented as mean ± SD. Multiple Student’s *t*-test was used for comparison between two groups. Analysis of variance was used for comparisons among multiple groups. *P* values of <0.05 were considered statistically significant. Analyses were conducted using GraphPad Prism Software (version 8.3.0).

## Data Availability

The authors confirm that the data supporting the findings of this study are available in the article and its supplementary material.

## SUPPLEMENTAL MATERIAL

Figure S1 mbio.02944-25-s0001.pdf Significantly enriched GO terms.

Table S1 mbio.02944-25-s0002.pdf Strains and plasmids used in this study.

Table S2 mbio.02944-25-s0003.pdf PCR primers used in this study.

ASM does not own the copyrights to Supplemental Material that may be linked to, or accessed through, an article. The authors have granted ASM a non-exclusive, world-wide license to publish the Supplemental Material files. Please contact the corresponding author directly for reuse.
