# Supplementary material for: TetR- and LysR-type transcriptional regulators mediate multilayered control of T3SS1 by Vibrio parahaemolyticus quorum sensing
Source: mBio. 2025 Nov 12;16(12):e02944-25. doi: 10.1128/mbio.02944-25 (PMC12691590; doi:10.1128/mbio.02944-25)
Supplement: Figure S1 — Significantly enriched GO terms. [file mbio.02944-25-s0001.pdf]

Figure S1

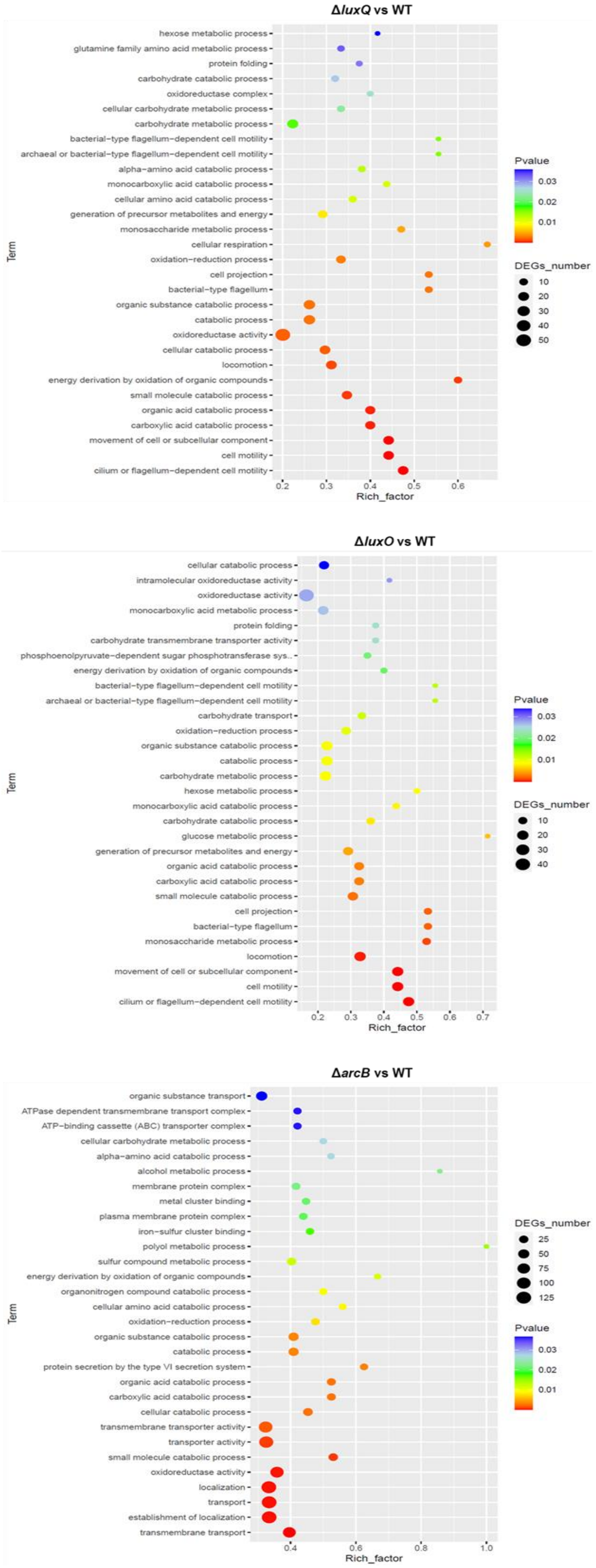
ΔluxQ vs WT
hexose metabolic process
glutamine family amino acid metabolic process
protein folding
carbohydrate catabolic process
oxidoreductase complex
cellular carbohydrate metabolic process
carbohydrate metabolic process
bacterial−type flagellum−dependent cell motility
archaeal or bacterial−type flagellum−dependent cell motility
alpha−amino acid catabolic process
monocarboxylic acid catabolic process
cellular amino acid catabolic process
generation of precursor metabolites and energy
monosaccharide metabolic process
cellular respiration
oxidation−reduction process
cell projection
bacterial−type flagellum
organic substance catabolic process
catabolic process
oxidoreductase activity
cellular catabolic process
locomotion
energy derivation by oxidation of organic compounds
small molecule catabolic process
organic acid catabolic process
carboxylic acid catabolic process
movement of cell or subcellular component
cell motility
cilium or flagellum−dependent cell motility
Term
0.2 0.3 0.4 0.5 0.6
Rich_factor
Pvalue
0.03
0.02
0.01
DEGs_number
10
20
30
40
50
ΔluxO vs WT
cellular catabolic process
intramolecular oxidoreductase activity
oxidoreductase activity
monocarboxylic acid metabolic process
protein folding
carbohydrate transmembrane transporter activity
phosphoenolpyruvate−dependent sugar phosphotransferase sys..
energy derivation by oxidation of organic compounds
bacterial−type flagellum−dependent cell motility
archaeal or bacterial−type flagellum−dependent cell motility
carbohydrate transport
oxidation−reduction process
organic substance catabolic process
catabolic process
carbohydrate metabolic process
hexose metabolic process
monocarboxylic acid catabolic process
carbohydrate catabolic process
glucose metabolic process
generation of precursor metabolites and energy
organic acid catabolic process
carboxylic acid catabolic process
small molecule catabolic process
cell projection
bacterial−type flagellum
monosaccharide metabolic process
locomotion
movement of cell or subcellular component
cell motility
cilium or flagellum−dependent cell motility
Term
0.2 0.3 0.4 0.5 0.6 0.7
Rich_factor
Pvalue
0.03
0.02
0.01
DEGs_number
10
20
30
40
ΔarcB vs WT
organic substance transport
ATPase dependent transmembrane transport complex
ATP−binding cassette (ABC) transporter complex
cellular carbohydrate metabolic process
alpha−amino acid catabolic process
alcohol metabolic process
membrane protein complex
metal cluster binding
plasma membrane protein complex
iron−sulfur cluster binding
polyol metabolic process
sulfur compound metabolic process
energy derivation by oxidation of organic compounds
organonitrogen compound catabolic process
cellular amino acid catabolic process
oxidation−reduction process
organic substance catabolic process
catabolic process
protein secretion by the type VI secretion system
organic acid catabolic process
carboxylic acid catabolic process
cellular catabolic process
transmembrane transporter activity
transporter activity
small molecule catabolic process
oxidoreductase activity
localization
transport
establishment of localization
transmembrane transport
Term
0.4 0.6 0.8 1.0
Rich_factor
DEGs_number
25
50
75
100
125
Pvalue
0.03
0.02
0.01

Fig S1. Bubble plot displaying significantly enriched Gene Ontology (GO) terms (p-value < 0.05). GO annotation of the DEGs was used to understand the functional classification of the DEGs. Bubble size: Number of genes associated with the term. Color gradient: Enrichment significance (p-value), where darker/redder indicates higher significance. Y-axis: Enriched GO terms (grouped by Biological Process, Molecular Function, or Cellular Component). X-axis: Gene Ratio (proportion of genes in the term relative to the background).
