# Supplementary material for: TetR- and LysR-type transcriptional regulators mediate multilayered control of T3SS1 by Vibrio parahaemolyticus quorum sensing
Source: mBio. 2025 Nov 12;16(12):e02944-25. doi: 10.1128/mbio.02944-25 (PMC12691590; doi:10.1128/mbio.02944-25)
Supplement: Table S1 — Strains and plasmids used in this study. [file mbio.02944-25-s0002.pdf]

Supplementary table 1 (Table S1). Strains and plasmids used in this study.

Strains and plasmids	Relevant characteristics	Reference
V. parahaemolyticus RIMD2210633	Clinical isolate O3: K6	(1)
Δ*luxQ*	*luxQ* knockout	Lab collection
Δ*arcB*	*arcB* knockout	Lab collection
Δ*luxO*	*luxO* knockout	Lab collection
Δ*vcrD1*	*vcrD1* knockout	Lab collection
Δ*exsA*	*exsA* knockout	Lab collection
Δ*aphA*	*aphA* knockout	This study
Δ*opaR*	*opaR* knockout	This study
Δ*vltR*	*vltR* knockout	This study
Δ*tftR*	*tftR* knockout	This study
Δ*aphA*Δ*vltR*	*aphA vltR* knockout	This study
Δ*aphA*Δ*tftR*	*aphA tftR* knockout	This study
Δ*opaR*Δ*vltR*	*opaR vltR* knockout	This study
Δ*opaR*Δ*tftR*	*opaR tftR* knockout	This study
Δ*vltR*:pBBR1MCS-1-*vltR*	*vltR* knockout complemented with *vltR* by pBBR1MCS-1	This study
Δ*tftR*:pBBR1MCS-1-*tftR*	*tftR* knockout complemented with *tftR* by pBBR1MCS-1	This study
Δ*opaR*:pBBR1MCS-1-*opaR*	*opaR* knockout complemented with *opaR* by pBBR1MCS-1	This study
Δ*aphA*:pBBR1MCS-1-*aphA*	*aphA* knockout complemented with *aphA* by pBBR1MCS-1	This study
Δ*opaR*:pMMB207-*opaR*-Flag	*opaR* knockout complemented with *opaR*-Flag by pMMB207	This study
Δ*aphA*Δ*opaR*:pMMB207-*opaR*-Flag	*aphA opaR* knockout complemented with *opaR*-Flag by pMMB207	This study
Δ*tftR*Δ*opaR*:pMMB207- *opaR*-	*tftR opaR* knockout complemented with *opaR*-	This

Flag	Flag by pMMB207	study
RIMD: pHRP309-P_{exsB}-*lacZ*	WT complemented with lacZ reporter vector pHRP309 carrying the *exsB* promoter	This study
Δ*tftR*: pHRP309-P_{exsB}-*lacZ*	*tftR* knockout complemented with lacZ reporter vector pHRP309 carrying the *exsB* promoter	This study
Δ*vltR*: pHRP309-P_{exsB}-*lacZ*	*vltR* knockout complemented with lacZ reporter vector pHRP309 carrying the *exsB* promoter	This study
Δ*aphA*: pHRP309-P_{exsB}-*lacZ*	*aphA* knockout complemented with lacZ reporter vector pHRP309 carrying the *exsB* promoter	This study
Δ*opaR*: pHRP309-P_{exsB}-*lacZ*	*opaR* knockout complemented with lacZ reporter vector pHRP309 carrying the *exsB* promoter	This study
Δ*aphA*: pHRP309-P_{opaR}-*lacZ*	*aphA* knockout complemented with lacZ reporter vector pHRP309 carrying the *opaR* promoter	This study
Δ*opaR*: pHRP309-P_{opaR}-*lacZ*	*opaR* knockout complemented with lacZ reporter vector pHRP309 carrying the *opaR* promoter	This study
Δ*tftR*: pHRP309-P_{opaR}-*lacZ*	*tftR* knockout complemented with lacZ reporter vector pHRP309 carrying the *opaR* promoter	This study
Δ*tftR*Δ*aphA*: pHRP309-P_{opaR}-*lacZ*	*tftR aphA* knockout complemented with lacZ reporter vector pHRP309 carrying the *opaR* promoter	This study
Δ*tftR*Δ*opaR*: pHRP309-P_{opaR}-*lacZ*	*tftR opaR* knockout complemented with lacZ reporter vector pHRP309 carrying the *opaR* promoter	This study
RIMD: pHRP309-P_{vltR} -*lacZ*	WT complemented with lacZ reporter vector pHRP309 carrying the *vltR* promoter	This study
Δ*vltR*: pHRP309-P_{vltR} -*lacZ*	*vltR* knockout complemented with lacZ reporter vector pHRP309 carrying the *vltR* promoter	This study
RIMD: pHRP309-P_{tftR}-*lacZ*	WT complemented with lacZ reporter vector pHRP309 carrying the *tftR* promoter	This study
Δ*tftR*: pHRP309-P_{tftR}-*lacZ*	*tftR* knockout complemented with lacZ reporter vector pHRP309 carrying the *tftR* promoter	This study
E.coli S17−1*λpir*	*thi pro hsdR hsdM*$^{+}$ *recA* RP4−2-Tc::MuKm::Tn7 *λpir*	(2)
S17:pDM4-Δ*aphA*	S17−1 strain carrying the plasmid pDM4_Δ*aphA*	This study
S17:pDM4-Δ*opaR*	S17−1 strain carrying the plasmid pDM4_Δ*opaR*	This study
S17:pDM4-Δ*vltR*	S17−1 strain carrying the plasmid pDM4_Δ*vltR*	This study
S17:pDM4-Δ*tftR*	S17−1 strain carrying the plasmid pDM4_Δ*tftR*	This study
S17:pDM4-Δ*aphA*Δ*vltR*	S17−1 strain carrying the plasmid pDM4-Δ*aphA*Δ*vltR*	This study
S17:pDM4-Δ*aphA*Δ*tftR*	S17−1 strain carrying the plasmid pDM4-	This

	Δ*aphA*Δ*tftR*	study
S17:pDM4-Δ*opaR*Δ*tftR*	S17−1 strain carrying the plasmid pDM4-Δ*opaR*Δ*tftR*	This study
S17:pBBR1MCS-1_*vltR*	S17−1 strain carrying the plasmid pBBR1MCS-1_*vltR*	This study
S17:pBBR1MCS-1_*tftR*	S17−1 strain carrying the plasmid pBBR1MCS-1_*tftR*	This study
S17:pBBR1MCS-1_*opaR*	S17−1 strain carrying the plasmid pBBR1MCS-1_*opaR*	This study
S17:pBBR1MCS-1_*aphA*	S17−1 strain carrying the plasmid pBBR1MCS-1_*aphA*	This study
S17:pMMB207_*opaR* Flag	S17−1 strain carrying the plasmid pMMB207_*opaR* -Flag	This study
S17:pHRP309-P_{exsB}-*lacZ*	S17−1 strain carrying the plasmid pHRP309-P_{exsB}-*lacZ*	This study
S17: pHRP309-P_{opaR}-*lacZ*	S17−1 strain carrying the plasmid pHRP309-P_{opaR}-*lacZ*	This study
S17: pHRP309-P_{vltR} -*lacZ*	S17−1 strain carrying the plasmid pHRP309-P_{vltR} -*lacZ*	This study
S17: pHRP309-P_{tftR}-*lacZ*	S17−1 strain carrying the plasmid pHRP309-P_{tftR}-*lacZ*	This study
E. coli BL21(DE3)	F- *ompT hsdSB* (rB- mB-) *gal dcm* (DE3)	
DE3:pCold-*aphA*	DE3 strain carrying the plasmid pCold-*aphA*	This study
DE3:pCold-*opaR*	DE3 strain carrying the plasmid pCold-*opaR*	This study
DE3:pCold-*tftR*	DE3 strain carrying the plasmid pCold-*tftR*	This study
DE3:pCold-*vltR*	DE3 strain carrying the plasmid pCold-*vltR*	This study
Plasmids	Relevant characteristics	Reference
pDM4 vector	Cm^r, suicide vector with an R6K origin (pir-requiring) and *sac*BR of *Bacillus subtilis*	(3)
pMMB207 vector	Cm^r, RSF1010 derivative, *IncQ lacI*^{q} Tac *ori*T	(4)
pBBR1MCS-1 vector	Cm^r, Broad-host range vector, *lacZα rep mob*	(5)
pCold TF DNA Vector	Amp, Trigger Factor, *cspA*, TEE, thrombin, factor Xa	(6)
pDM4-Δ*aphA*	pDM4 containing the flanking region sequences of *aphA*	This study
pDM4-Δ*opaR*	pDM4 containing the flanking region sequences of *opaR*	This study
pDM4-Δ*vltR*	pDM4 containing the flanking region sequences of *vltR*	This study

pDM4-Δ*tftR*	pDM4 containing the flanking region sequences of *tftR*	This study
pDM4-Δ*aphA*Δ*vltR*	pDM4 containing the flanking region sequences of *aphA* and *vltR*	This study
pDM4-Δ*aphA*Δ*tftR*	pDM4 containing the flanking region sequences of *aphA and tftR*	This study
pDM4-Δ*opaR*Δ*tftR*	pDM4 containing the flanking region sequences of *opaR* and *tftR*	This study
pBBR1MCS-1_*vltR*	pBBR1MCS-1 containing the *vltR*	This study
pBBR1MCS-1_*tftR*	pBBR1MCS-1 containing the *tftR*	This study
pBBR1MCS-1_*opaR*	pBBR1MCS-1 containing the *opaR*	This study
pBBR1MCS-1_*aphA*	pBBR1MCS-1 containing the *aphA*	This study
pMMB207_*opaR*-Flag	pMMB207 containing the *opaR*-Flag	This study
pHRP309-P_{exsB}-*lacZ*	pHRP309 containing the P_{exsB}-*lacZ*	This study
pHRP309-P_{opaR}-*lacZ*	pHRP309 containing the P_{opaR}-*lacZ*	This study
pHRP309-P_{vltR} -*lacZ*	pHRP309 containing the P_{vltR} -*lacZ*	This study
pHRP309-P_{tftR} -*lacZ*	pHRP309 containing the P_{tftR} -*lacZ*	This study
pCold-*aphA*	pCold-TF containing the *aphA*	This study
pCold-*opaR*	pCold-TF containing the *opaR*	This study
pCold-*tftR*	pCold-TF containing the *tftR*	This study
pCold-*vltR*	pCold-TF containing the *vltR*	This study

REFERENCES

1. Makino K, Oshima K, Kurokawa K, Yokoyama K, Uda T, Tagomori K, Iijima Y, Najima M, Nakano M, Yamashita A, Kubota Y, Kimura S, Yasunaga T, Honda T, Shinagawa H, Hattori M, Iida T. 2003. Genome sequence of *Vibrio parahaemolyticus*: a pathogenic mechanism distinct from that of *V cholerae*. Lancet 361:743-749.
2. Milton DL, Norqvist A, Wolf-Watz H. 1992. Cloning of a metalloprotease gene involved in the virulence mechanism of *Vibrio anguillarum*. J Bacteriol 174:7235-44.
3. Milton DL, O'Toole R, Horstedt P, Wolf-Watz H. 1996. Flagellin A is essential for the virulence of *Vibrio anguillarum*. J Bacteriol 178:1310-9.
4. Morales VM, Bäckman A, Bagdasarian M. 1991. A series of wide-host-range low-copy-number vectors that allow direct screening for recombinants. Gene 97:39-47.
5. Kovach ME, Elzer PH, Hill DS, Robertson GT, Farris MA, Roop RM, 2nd, Peterson KM. 1995. Four new derivatives of the broad-host-range cloning vector pBBR1MCS, carrying different antibiotic-resistance cassettes. Gene 166:175-6.
6. Qing G, Ma LC, Khorchid A, Swapna GV, Mal TK, Takayama MM, Xia B, Phadtare S, Ke H, Acton T, Montelione GT, Ikura M, Inouye M. 2004. Cold-shock induced high-yield protein production in *Escherichia coli*. Nat Biotechnol 22:877-82.
