# Supplementary material for: TetR- and LysR-type transcriptional regulators mediate multilayered control of T3SS1 by Vibrio parahaemolyticus quorum sensing
Source: mBio. 2025 Nov 12;16(12):e02944-25. doi: 10.1128/mbio.02944-25 (PMC12691590; doi:10.1128/mbio.02944-25)
Supplement: Table S2 — PCR primers used in this study. [file mbio.02944-25-s0003.pdf]

**Supplementary table 2 (Table S2). PCR primers used in this study.**

| | | |
|---|---|---|
| Δ*aphA*-1F-Xba I | cgcatgcaagatctatctagaAAAATAACCATTCGTAATACAAAAGGC | Construction of suicide plasmid pDM4_Δ*aphA* for *aphA* knockout |
| Δ*aphA*-1R | TTTTCGTTGTCTCTAAGTAAAAGGAAATGTCTTCAATCCAAATGGTCAATAAAC | |
| Δ*aphA*-2F | GTTTATTGACCATTTGGATTGAAGACATTTCCTTTTACTTAGAGACAACGAAAA | |
| Δ*aphA*-2R-Xba I | cactagtggggcccttctagaACGTAAAGTGGATGCTGGTGC | |
| Cold-*aphA*-F-SacI | ggcatatggagctcGATTACAAGGATGACGATGACAAGATGTCATTACCACACGTAATC | Construction of recombinant plasmid pCold-*aphA* |
| Cold-*aphA*-R-EcoRI | caggtcgacaagcttgaattcCCAATCACTTCAAGTTCTGTTAGG | |
| Δ*tftR* -1F-Xba I | cgcatgcaagatctatctagaTTTGATGGGCTCAACTACTGCA | Construction of suicide plasmid pDM4_Δ*tftR* for *tftR* knockout |
| Δ*tftR* -1R | CGAGCGACTATTTCGCCGATTTTCTTTTGTTGTGCTTCTCGG | |
| Δ*tftR* -2F | CCGAGAAGCACAACAAAAGAAAATCGGCGAAATAGTCGCTCG | |
| Δ*tftR* -2R-Xba I | cactagtggggcccttctagaTGCAATACGAGACACAATCGTTG | |
| Cold-*tftR*-F-SacI | ggcatatggagctcGATTACAAGGATGACGATGACAAGATGGCCCCGAGAAGCACA | Construction of recombinant plasmid pCold-*tftR* |
| Cold-*tftR*-R-EcoRI | caggtcgacaagcttgaattcTTTCGCCGACATACCAGCG | |
| pHRP309-$P_{tftR}$-SalI-F | ctgaatgaactgcaggtcgacCTCTGGTTGGGCAATTCCTG | Construction of recombinant plasmid pHRP309-$P_{tftR}$-*lacZ* |
| pHRP309-$P_{tftR}$_EcoRI-R | gtagaggatccccgggaattcCGATGCAAGATTCACATTTGCT | |
| Δ*opaR*-1F-Xba I | cgcatgcaagatctatctagaAGCACGCGCTAGGTCGGC | Construction of suicide plasmid pDM4_Δ*opaR* for *opaR* knockout |
| Δ*opaR*-1R | TGTTCGTGTTCAAATCTGAGCTTTAGTCTCTTTGCAATTGAGTCCATATCC | |
| Δ*opaR*-2F | GGATATGGACTCAATTGCAAAGAGACTAAAGCTCAGATTTGAACACGAACA | |
| Δ*opaR*-2R-Xba I | cactagtggggcccttctagaTGGCTTGGGTTGGTAAGACAG | |

| | | |
|---|---|---|
| Cold-*opaR*-F-SacI | ggcatatggagctcTACCCATACGACGTCCCAGACTACGCTATGGACTCAATTGCAAA | Construction of recombinant plasmid pCold-*opaR* |
| Cold- *opaR* -R-EcoRI | caggtcgacaagcttgaattcTTAGTGTTCGCGATTGTAGATGC | |
| pHRP309-$P_{opaR}$ -SalI-F | ctgaatgaactgcaggtcgacCACTGCCTTGGTAACGCTCTG | Construction of recombinant plasmid pHRP309-$P_{opaR}$-*lacZ* |
| pHRP309-$P_{opaR}$ _EcoRI-R | gtagaggatccccgggaattcCAATATCTGCGTGACCACCACG | |
| Δ*vltR* -1F-Xba I | cgcatgcaagatctatctagaCGTAATGACAACGTGACCGATT | Construction of suicide plasmid pDM4_Δ*vltR* for *vltR* knockout |
| Δ*vltR* -1R | ATGCTAAAAGAACGTTAGTTTGCAATTTGAATATCCAGATTAATCGCCA | |
| Δ*vltR*-2F | TGGCGATTAATCTGGATATTCAAATTGCAAACTAACGTTCTTTTAGCAT | |
| Δ*vltR*-2R-Xba I | cactagtggggcccttctagaTCACATCAAAGCGAAGTGTGTCA | |
| Cold-*vltR*-F-SacI | ggcatatggagctcTACCCATACGACGTCCCAGACTACGCTATGGCGATTAATCTGGAT | Construction of recombinant plasmid pCold-*vltR* |
| Cold-*vltR*-R-EcoRI | caggtcgacaagcttgaattcGTTTGCAATCACGTGTTTAATCTTATC | |
| pHRP309-$P_{vltR}$-SalI-F | ctgaatgaactgcaggtcgacCCACTTGATTACATACGCAATCAAC | Construction of recombinant plasmid pHRP309-$P_{vltR}$ -*lacZ* |
| pHRP309-$P_{vltR}$_EcoRI-R | gtagaggatccccgggaattcAAGCCATGTTTTGTTTGCACAA | |
| pHRP309-$P_{exsB}$-SalI-F | ctgaatgaactgcaggtcgacATTCGATATCGTGGGGTGTGTT | Construction of recombinant plasmid pHRP309-$P_{exsB}$-*lacZ* |
| pHRP309-$P_{exsB}$_EcoRI-R | gtagaggatccccgggaattcTAACATTAAACCGATAAGACCGGC | |
| qPCR-*tftR*-F | CAAGGGCATTTGCGTTGGTT | qRT-PCR |
| qPCR-*tftR*-R | CGACATACCAGCGGACAAGT | qRT-PCR |
| qPCR-*opaR*-F | ATTTGCACGCCGTGGTATTG | qRT-PCR |
| qPCR-*opaR*-R | AGCTCGATCATCGCATTGGT | qRT-PCR |
| qPCR-*vltR*-F | GAACACCCAGCGATGATCCA | qRT-PCR |
| qPCR-*vltR*-R | TTTAACGGTGCGTGTTGCTG | qRT-PCR |
| qPCR-*aphA*-F | CTGGAAAGCAAGCCACCAAC | qRT-PCR |
| qPCR-*aphA*-R | ATTCTCCAAGAGCGCTACGG | qRT-PCR |
| qPCR-*cytR*-F | TACCTCGTACTGCTCGGTGA | qRT-PCR |
| qPCR-*cytR*-R | ACATCAAATGGCAGGTCGGT | qRT-PCR |
| qPCR-*dhaR*-F | CCTGGGATGAAGAAAGCCGT | qRT-PCR |

| | | |
|---|---|---|
| qPCR-*dhaR*-R | CTTGCACCCGACTCAAGACT | qRT-PCR |
| qPCR-*exsA*-F | ATGAAAACGCTGGAGGAGCA | qRT-PCR |
| qPCR-*exsA*-R | ATAGAGGACTTCGCCCCCTT | qRT-PCR |
| qPCR-*lexA*-F | CGCACTACCAAGTTGACCCT | qRT-PCR |
| qPCR-*lexA*-R | TTGTGTTTTGTGCACGGCAA | qRT-PCR |
| qPCR-*RS18740*-F | CAACTGCCCAAAATTGCCGA | qRT-PCR |
| qPCR-*RS18740*-R | TGGTGGCCACTGAAATGTGT | qRT-PCR |
| qPCR-*RS19765*-F | GCATTGTGCCACATTACGCA | qRT-PCR |
| qPCR-*RS19765*-R | GAGAGCCGACCAATTGACCA | qRT-PCR |
| qPCR-*exsB*-F | ACCATCCTCCAGTTTCGA | qRT-PCR |
| qPCR-*exsB*-R | GTTTCCCTATCACTTTCATTTC | qRT-PCR |
| qPCR-*vcrD1*-F | GCCTGTTCTGGTGGTTTC | qRT-PCR |
| qPCR-*vcrD1*-R | CATCCCTACCCGTCCAAG | qRT-PCR |
| qPCR-*rpoA*-F | TGGCTTTGGTCATACTCT | qRT-PCR |
| qPCR-*rpoA*-R | GTTGTCATCCGTTAGGTG | qRT-PCR |
| EMSA-*exsB*-F | TAATTGGTGTTCTGGCCAA | EMSA |
| EMSA-*exsB*-R | TAACATTAAACCGATAAG | EMSA |
| EMSA-*aphA*-F | CATTCGTAATACAAAAGG | EMSA |
| EMSA-*aphA*-R | TTCCAGAAGTAACCGATGCTAG | EMSA |
| EMSA-*opaR*-F | TCCATCGTGTTGCCGTAGC | EMSA |
| EMSA-*opaR*-R | CAATATCTGCGTGACCACCAC | EMSA |
| EMSA-*vltR*-F | AGCGGTTGTGATGGTGGCG | EMSA |
| EMSA-*vltR*-R | AAGCCATGTTTTGTTTGCA | EMSA |
| EMSA-*tftR*-F | ATTTCTCATTACCAAAGAAAGC | EMSA |
| EMSA-*tftR*-R | CGATGCAAGATTCACATTTG | EMSA |
